# The S100B/RAGE Axis in Alzheimer's Disease

**DOI:** 10.1155/2010/539581

**Published:** 2010-06-21

**Authors:** Estelle Leclerc, Emmanuel Sturchler, Stefan W. Vetter

**Affiliations:** ^1^Department of Pharmaceutical Sciences, North Dakota State University, Dept. 2665, P.O. Box 6050, Fargo, ND 58108-6050, USA; ^2^Department of Drug Discovery, The Scripps Research Institute, 130 Scripps Way, Jupiter, FL 33458, USA

## Abstract

Increasing evidence suggests that the small EF-hand calcium-binding protein S100B plays an important role in Alzheimer's disease. Among other evidences are the increased levels of both S100B and its receptor, the Receptor for Advanced Glycation Endproducts (RAGEs) in the AD diseased brain. The regulation of RAGE signaling by S100B is complex and probably involves other ligands including the amyloid beta peptide (A*β*), the Advanced Glycation Endproducts (AGEs), or transtheyretin. In this paper we discuss the current literature regarding the role of S100B/RAGE activation in Alzheimer's disease.

## 1. Introduction

Alzheimer's disease (AD) is the most common form of dementia in the elderly [1]. AD patients suffer from a progressive decline of cognitive functions that include language, personality, and memory impairments.

The pathological hallmarks of the disease are characterized by the presence of senile plaques (SPs), neurofibrillary tangles (NFTs), and severe gliosis in the cerebral cortex and the hippocampus [2]. Senile plaques result from the accumulation of extracellular amyloid-*β* (A*β*) fibrils [3] and contain elevated levels of zinc and copper ions [4]. Neurofibrillar tangles are mainly constituted of intracellular, abnormally phosphorylated tau protein [5–7]. AD brain is also characterized by increases in inflammatory responses, oxidative stress, dysregulation of calcium homeostasis [8], and by elevated levels of several S100 calcium-binding proteins namely S100B, S100A6, S100A9, and S100A12 [9–12]. 

Neurons, microglia, and endothelial cells, surrounding the senile plaques express higher levels of the receptor for advanced glycation endproducts (RAGEs) as the pathology progresses [13, 14]. Although its exact role in AD remains to be clearly established, RAGE appears to initiate several signal transduction cascades in response to ligands, related to AD including A*β*, AGEs, transthyretin, and S100 proteins. The present paper will focus and discuss the current knowledge on the role of S100B/RAGE axis in AD.

## 2. The Receptor for Advanced Glycation Endproducts

RAGE is an immunoglobulin-like cell surface receptor that is often described as a pattern recognition receptor due to the structural heterogeneity of its ligands. RAGE was initially identified as receptor for the advanced glycation endproducts (AGEs) [15, 16]. AGEs are formed by nonenzymatic modification of proteins or lipids by reducing carbohydrates, are highly heterogeneous (reviewed in [17]), and are often found elevated at sites of inflammation where they can trigger RAGE-dependent oxidative stress and NF-*κ*B activation. NF-*κ*B activation leads to increased RAGE expression because of the presence of NF-*κ*B response elements within the promotor region of RAGE [18]. Activation of RAGE in turn results in sustained NF-*κ*B activation [19]. Positive feedback loops between RAGE, oxidative stress, and inflammation can thus develop [20]. In this view high levels of AGEs have been found at site of inflammation and colocalize with neurofibrillar tangles and senile plaques in AD brain [21–23].

A second group of RAGE ligand is formed by amyloid-forming proteins or peptides such as A*β* peptide [13], and transthyretin (TTR) [24]. The amyloid *β*-peptide results from amyloid precursor protein (APP) processing by the beta and gamma secretases. A*β* accumulation in the brain plays a key role in the development of the disease [13, 25]. RAGE has been shown to mediate the transport of A*β* through the neuronal cell membrane and blood brain barrier [13, 26, 27]. In contrast, TTR has been suggested to have a protective effect in AD by binding to A*β* in a chaperone-like manner [28]. 

RAGE can also be activated by amphoterin (High Mobility Group Box 1, HMGB1) that plays a role in neuronal development and cancer [29]. Although it also plays a role in inflammation, we will not discuss the putative role of amphoterin in AD in this paper [30].

Another group of RAGE ligand is constituted by the S100 proteins. S100 proteins are small EF-hand calcium-binding proteins that regulate calcium homeostasis and modulate various enzymes involved in cellular functions such as cell growth, differentiation, and metabolism (reviewed in [31–33]). Twenty one members of S100 proteins have been described [34]. They all share high amino acid and structural homologies. Among them S100B, S100A6, S100A9 and S100A12 have been linked to Alzheimer's Disease [9–12]. 

**Figure 1:**
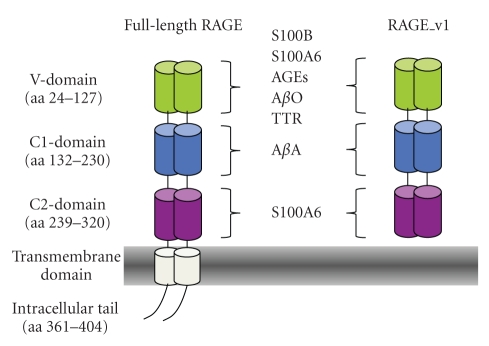
Schematic representation of the two main RAGE isoforms, full-length RAGE and RAGE_v1. Full-length RAGE is an immunoglobulin like receptor with one variable-like domain (V) and two constant-like domains (C) comprising residues. A short transmembrane domain anchors RAGE to the cell surface. A 41 residues intracellular tail is critical for signal transduction. RAGE_v1 does not possess the transmembrane domain and the intracellular tail. It is soluble in the circulation and plays the role of decoy to antagonize the activation of full-length RAGE by its ligands. A soluble form of RAGE can also be generated by proteolysis. S100B, AGEs A*β* oligomers, and TTR bind to RAGE V domain. A*β* aggregates binds to RAGE C1 domain. S100A6 binds both to the V and C2 domain but exerts its cellular effects preferentially through the C2 domain. The exact oligomerization states of full-length RAGE and RAGE_v1 are currently unknown. RAGE is arbitrarily represented as a dimer.

Various alternatively spliced isoforms of RAGE exist [16, 35]. The two prevalent isoforms appear to be the full-length RAGE (RAGE) and the secreted isoform RAGE_v1 [36]. Full-length RAGE is composed of an extracellular part (314 aa), a single transmembrane spanning helix (27 aa), and a short cytosolic domain (41 aa) (Figure 1) [16]. The extracellular part of RAGE contains an Ig-like V-domain (residues 24–127) and two constant Ig-like C type domains frequently referred to as C1 (residues 132–230) and C2 domains (residues 239–320). RAGE possesses two N-glycosylation sites, one adjacent to the V-domain (residue 26) and the second one within the V domain (residue 81) (Figure 1) [16, 37]. Recent studies suggest that glycosylation may modulate the interaction of certain AGEs with RAGE [38, 39]. The RAGE_v1 splice isoform lacks the transmembrane and cytoplasmic portion and is released in the extracellular space (Figure 1) [36, 40–42]. The distribution and relative expression of the different RAGE isoforms are tissue specific. The full-length RAGE isoform is present at low levels in most adult tissues but at relatively high levels in lungs [43]. The truncated variant RAGE_v1 appears to be the prevalent isoform in endothelial cells and in human brain (Figure 1) [41, 44]. Interestingly, the soluble form of RAGE (sRAGE) can also be produced by proteolytic cleavage [45–47]. sRAGE produced either by splicing or shedding has been suggested to play the role of a decoy that interacts with free circulating RAGE ligand. RAGE_v1 expression is reduced in hippocampal neurons of AD patients. This could potentially lead to a sustained RAGE activation [48, 49]. In this view, sRAGE formed as a result of proteolysis could prevent A*β* peptide transport across the blood brain barrier and protect against Alzheimer's disease [50]. In the last five years, soluble RAGE has emerged as a new biomarker with potential clinical and therapeutic applications (reviewed in [51, 52]) and polypeptides based on RAGE_v1 are currently tested in clinical trials for their therapeutic effects against deleterious effects triggered by RAGE activation by its ligands.

However, the role of sRAGE and its regulation appears to be very complex. Indeed recent studies aiming at comparing the concentration of sRAGE in the serum of patients versus controls in various pathophysiological conditions have shown both negative and positive correlation between the concentration of sRAGE and the severity of the disease ([53, 54] and reviewed in [55]). Moreover, blocking RAGE function might not be beneficial in all pathologies. Indeed RAGE has been shown to modulate the regeneration of peripheral nerves in a mouse model of axotomy and blockade of RAGE signaling using sRAGE resulted in impaired regeneration in these animals [56–58]. Although a large number of studies with rodent models of human diseases have demonstrated the short-term benefit of treatment with sRAGE (reviewed in [52]), the long-term effects of such treatments remain also to be studied. The role of sRAGE as a decoy that would neutralize the excess of RAGE ligands also needs to be reconsidered at the view at recent studies showing very low concentrations (10–50 pM) of sRAGE in the serum of both patients and healthy individuals [53–55]. 

In order to understand the role of RAGE in the various RAGE-related pathologies including AD, it is important to understand how the different RAGE ligands interact with the receptor. Binding and activation of RAGE by S100B was first demonstrated in HUVEC cells [59]. In recent years, our laboratory and others have studied in detail the interaction of S100B with RAGE. S100B interacts preferentially with the V domain of RAGE and might involve multimerization of the receptor [60–62]. The V domain of RAGE is also the binding site of AGEs and TTR [63–66]. 

A*β*-RAGE interactions are more complex since A*β* exhibits several conformational states. A*β* is generated by proteolytic cleavage of the transmembrane *β*-amyloid precursor protein (APP) (reviewed in [67]). The resulting 1-40 or 1-42 amino acid A*β* peptides can form soluble oligomers (A*β*O), beta-sheet containing fibrils, and insoluble aggregates (A*β*A) [25, 68–74]. It is now believed that the synaptic dysfunction and neuronal death observed in AD patients are caused mainly by A*β* oligomers and A*β* fibrils [25, 71–73, 75–78]. We recently showed that the interaction of A*β* with RAGE is driven by conformational states of A*β*. Indeed A*β*O and A*β*A were found to bind to distinct domains of RAGE, the V-, and C1-domain, respectively. Furthermore, A*β*O RAGE interaction was found to modulate ERK activity and to induce neuronal death [25].

Although S100B, AGEs, and A*β*O interact with the V domain of the receptor, it is currently not known if they interact within the same region of the V domain. Future studies will answer this question.

## 3. RAGE in Alzheimer's Disease

RAGE is up-regulated in the brain of Azheimer's disease and triggers the generation of proinflammatory cytokines at the blood brain barrier [13, 27]. The role of RAGE in AD has been demonstrated in cell culture and in animal models. In various cell types that include neurons, endothelial cells, and microglia, engagement of RAGE by A*β* can lead to the formation of reactive oxygen species (ROS), the activation of NF-*κ*B, or the expression of cell adhesion molecules mediating the recruitment of inflammatory cells [79, 80]. Neurons overexpressing RAGE showed higher susceptibility to A*β*-induced cell death than control cells [81]. Several models of transgenic mice have been used to demonstrate the role of RAGE in AD. The double transgenic RAGE/APP mouse model combines the overexpression of RAGE with the expression of mutants of APP [82, 83]. RAGE/APP mice show impaired spatial learning and memory capabilities, reduced basal synaptic transmission and long-term potentiation (LTP) compared to their single transgenic littermates. At the cellular level, these mice show reduced density of cholinergic fibers and synapses, characteristics often associated with AD-like pathology [83]. At the molecular level, RAGE/APP mice show enhanced activation of inflammation and stress-related MAP kinases and of the transcription factors NF-*κ*B [83]. Despite these evidences the role of RAGE in Alzheimer's disease is still to be understood in detail. Indeed, recent experiments performed on RAGE (-/-) arcAbeta double transgenic animals showed that RAGE deletion could not prevent the decline in cognitive performance of the mice nor the age-related cerebral accumulation of A*β* peptides [84]. These discrepancies may be due to differences in the mouse models used in the distinct studies. The Arc mutation is characterized by a change in amino acid within the A*β* peptide sequence and thus may generate distinct peptide conformations that have less or no affinity for RAGE. Interestingly, the arcBeta transgenic mice showed reduced clearance of A*β* accross blood vessels [85]. This could reflect a decrease in the binding capacity of the arcAbeta for another of its receptor, LRP that has been shown to mediate brain efflux of A*β* [86].

## 4. S100B

S100B is a member of the S100 protein family mainly expressed in the CNS [87]. Animal studies using S100B transgenic mice revealed that S100B plays important roles in spatial and fear memory, learning capabilities, and epileptogenesis [88–90].

Unlike other members of the S100 protein family, the gene of S100B is located on human chromosome 21 [91, 92]. S100B possesses two Ca^2+^-binding sites of the EF-hand type, defined as a helix-loop-helix motif connected by a central hinge region. The C-terminal domain contains the classical EF-hand with a canonical 12 amino acid Ca^2+^-binding loop whereas the N-terminal domain contains the S100B specific 14 amino acid Ca^2+^-binding loop [93, 94]. S100B binds two calcium ions per subunit with moderate affinity (2–20 *μ*M) [95]. Binding of calcium to the EF-hands triggers structural changes that allow the interaction with target proteins [32, 96]. Besides calcium, S100B also binds zinc (K_D_ = 0.1–1 *μ*M) and copper (K_D_ = 2.2 *μ*M), two metal ions highly abundant in senile plaques [32, 96–98]. Interestingly, binding of zinc to S100B results in higher affinity for both calcium and S100B's target proteins. The extracellular function of S100B may thus be altered in the brain of AD patients due to the high levels of zinc and copper [99].

S100B interacts with various intracellular targets. These targets have been extensively described in previous reviews [98, 100, 101]. S100B interacts with elements of the cytoskeleton (microtubules, type III intermediate filaments), with enzymes of the glycolytic pathway (fructose 1,6-bisphosphate aldolase, phosphoglucomutase), and with the tumor suppressor p53. S100B also regulates calcium homeostasis, protein phosphorylation and degradation [100]. S100B is mainly found as homodimer but can also form active tetramers, or hexamers exhibiting distinct functions [61, 102–104]. Furthermore, S100B is also able to interact with S100A1. This protein complex exhibits distinct physiological functions compared to S100B or S100A1 homodimers [32, 61, 102–105]. 

Besides its known intracellular function, S100B can also be secreted in the extracellular space where it acts as a cytokine. The secretion of S100B occurs via both the classical endoplasmic reticulum-Golgi pathway and an alternative pathway involving cytoskeletal tubulin [106, 107]. 

High levels of extracellular S100B have been detected in various clinical conditions that include brain trauma, ischemia and neurodegenerative, and inflammatory and psychiatric diseases [108, 109]. S100B is also a well-established prognostic marker for melanoma and high serum concentration of S100B correlate with poor prognosis [110, 111]. 

**Figure 2:**
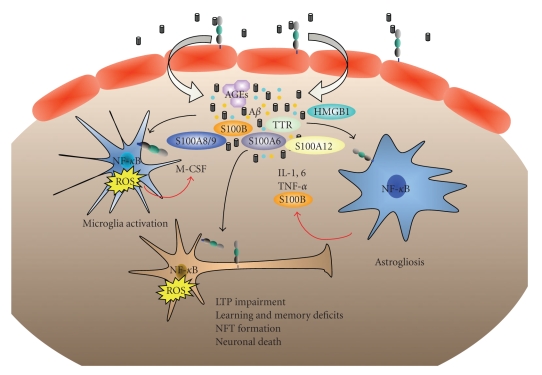
Crosstalk between RAGE and its ligands in Alzheimer's disease. RAGE mediates A*β* brain influx and accumulation. A*β* directly or indirectly triggers dysregulation of calcium homeostasis thereby activating the S100 proteins. RAGE-mediated activation of glia cells results in the activation of NF-*κ*B driven gene transcription, and the release of inflammatory cytokines such as IL-1, IL-6, TNF-*α*, IL-1*β*, M-CSF and S100B. The brain of AD patients becomes the site of intense inflammation and oxidative stress that facilitates formation of AGEs. S100B, A*β* and AGEs as well as other RAGE ligands including TTR, HMGB1, S100A6, S100A8/A9, and S100A12 accumulate in the brain during the course of the disease. Secreted S100B and chronic RAGE activation trigger several AD-associated neuropathological features including microglia activation, the production of reactive oxygen species (ROS), neurite degeneration, NFT formation, and neuronal apoptosis ultimately leading to memory impairment.

In the brain, S100B is actively secreted from astrocytes in the extracellular medium (Figure 2) [112]. S100B release is driven by the developmental stage of the astrocytes [112], and metabolic stress (oxygen, serum, or glucose deprivation) [113]. S100B can also be released in response to external stimuli such as glutamate [114], serotonin [115], the pro-inflammatory cytokines TNF-alpha [116] and IL-1beta [117], beta-amyloid peptides [118], 1-methyl-4-phenyl 1,2,3, and 6 tetrahydropyridine (MPTP) [119], forskolin, lysophosphatidic acid [120], and the plant natural antioxydants resveratrol and epicatechin [121, 122] and by the increase of calcium concentration [107]. 

Extracellular S100B has been shown to modulate the activity of neurons, microglia, astrocytes, monocytes, and endothelial cells (Figure 2). On neurons, S100B triggers trophic or toxic effects, depending on its concentration. Nanomolar concentration of S100B is neuroprotective, induces neurite outgrowth, and triggers glial cell proliferation in a RAGE dependent manner, whereas micromolar concentration of S100B is neurotoxic [120, 123–126]. At the molecular level, nanomolar concentration of S100B induces the upregulation of the antiapoptotic factor Bcl-2 resulting in neuroprotection. In contrast, when present in micromolar concentration S100B induces the up-regulation of caspase 3 through the activation of the oxidant stress-dependent MEK/ERK pathways, leading to apoptosis [126]. In addition, S100B can also modulate the toxicity of other extracellular molecules. In rat hippocampal neurons low concentration of S100B protects the cells against the toxic effect of N-methyl-D-aspartate, through the activation of NF-*κ*B and possibly through the engagment of RAGE [127]. S100B also protects astrocytes and microglia against toxicity of trimethyltin [128]. Similar protection is observed in LAN-5 neuroblastomas, in the presence of A*β* peptide [129]. Importantly, in these cells, higher concentration of S100B (micromolar) potentiates the toxicity of A*β* peptide. 

S100B activates astrocytes in an autocrine manner and triggers the release of TNF-*α* and IL-6, probably through the activation of RAGE [130] leading to cellular inflammation (Figure 2).

Extracellular S100B can also stimulate endothelial cells, resulting in perpetuated activation of NF-*κ*B and the up-regulation of vascular cell-adhesion molecule (VCAM-1) and the monocyte chemoattractant protein 1 (MCP-1) through the engagement of RAGE [59, 131]. Stimulation of endothelial cells by S100B results in adhesion and transendothelial migration of monocytes, leading to further inflammation in adjacent tissue [132]. Engagement of RAGE by S100B could thus contribute to the chronicity of inflammation observed to Alzheimer's disease [19].

## 5. S100B in Alzheimer's Disease

A role of S100B in AD is suggested by a large number of clinical studies showing elevated levels of S100B in the brain or cerebrospinal fluid of AD patients [9, 108, 133–137]. Furthermore, studies showed enhanced susceptibility to neuroinflammation and neuronal dysfunction after infusion of A*β* in transgenic mice overexpressing S100B supporting a role for S100B in AD [138]. Additional supporting evidence comes from recent studies using double transgenic mice over-expressing S100B and carrying mutation in APP (Tg2576/S100B) [139]. Over-expression of human S100B in these mice promotes brain inflammation as shown by astrogliosis and microgliosis and enhances A*β* generation from APP.

Evidence also suggests a role of S100B in the formation of neurofibrillar tangles. Hyperphosphorylated tau protein is the main component of neurofibrilar tangles. S100B binds directly to tau protein in the presence of calcium resulting in the inhibition of its phosphorylation by Ca^2+^/Calmodulin-dependent kinase II [140]. Intriguingly, extracellular S100B has also been found to promote RAGE-dependent hyperphosphorylation of tau protein through the modulation of the JNK and Wnt pathways [141]. Thus, S100B exhibits opposite effects depending on its localization. In AD patients S100B is actively released and may promote the hyperphosphorylation of tau protein and the development of neurofibrillary tangles in a RAGE dependent manner [129, 142, 143]. The secretion of S100B itself might be triggered by RAGE endocytosis [144, 145]. 

Thus, it is tempting to speculate that the role of S100B in Alzheimer's disease is mediated by RAGE and numerous studies mentioned in this paper support this hypothesis. Targeting specifically RAGE/S100B interaction in the brain might be beneficial to AD patients. Another interesting therapeutic approach may be to inhibit the binding of both S100B and A*β* to the V domain of RAGE using specific antibodies or small molecules.

## 6. Other RAGE Ligands in Alzheimer's Disease

Besides S100B, RAGE can also be engaged by other ligands that are all relevant in Alzheimer's disease. 

S100A6 is another member of the S100 protein family. S100A6 is upregulated in astrocytes of animal models and in patients with AD [10]. High levels of this protein were also found in the senile plaques of AD patients [10]. The exact role of S100A6 in AD is currently unknown but our recent studies suggest that S100A6 might play a role through RAGE. Indeed we recently showed that S100A6 interacts with both the V- and C2-domains of RAGE *in vitro*. However, in contrast to S100B, the cellular effects triggered by S100A6 appeared to occur via the C2-domain only [62]. Two other S100 family members, S100A8/A9 and S100A12 may play a role in AD as well by participating in inflammatory-mediated events contributing to neurodegeneration. High levels of S100A9 and S100A12 have been found in microglia of patients suffering from sporadic AD [11, 12]. As with S100B, the effects triggered by S100A8/A9 and S100A12 could involve RAGE. Indeed, these two cytokine-like S100 proteins have been shown to interact with RAGE and to trigger RAGE-dependent cellular signaling [59, 146, 147] leading to sustained inflammation [24, 28, 148–153]. Thus, RAGE can be engaged by distinct ligands associated with AD. 

Beside S100 proteins the senile plaques also contain elevated levels of AGEs, and TTR. The role of TTR in AD is suggested from both in vitro experiments and animal models studies [151–153]. RAGE interacts with both soluble and fibrillar TTR [149, 150]. TTR might have a protective effect in AD by binding to A*β* in a chaperone-like manner [28]. In AD settings, production of cytokines as a result of local inflammation would suppress TTR expression and reduce its protective role. However in other conditions TTR could also trigger NF-*κ*B activation through RAGE resulting in sustained inflammation and cellular stress [24, 150].

## 7. Synergistic Effects between RAGE Ligands

Recent cell-based experiments have shown synergistic effects between the different RAGE ligands. In cultured neurons, AGEs and A*β* act synergistically resulting in increased APP and RAGE expression [142]. In microglia, A*β* acts as an amplifier of the inflammatory response when cells are preactivated with AGEs [143]. In endothelial cells, only AGEs pretreated cells could respond to stimulation by S100A8/A9 [154]. As mentioned earlier, S100B can also potentiate the toxic effect of A*β* in LAN-5 neuroblastomas [129].

## 8. Conclusion

Alzheimer's disease is a complex disease involving many molecular partners including RAGE and S100B. Following the large number of promising studies where blockade of RAGE could reverse a large number of symptoms in animal models, RAGE became a well-pursued therapeutic target. We mentioned earlier in the paper that polypeptides based on the sequence of sRAGE were currently evaluated in clinical trials [155]. Small molecule compounds are also currently in phase 2 clinical trials (Pfizer: PF-04494700 [156]). Targeting RAGE would be beneficial to treat chronic RAGE-dependent pathologies. However, the recent studies on the role of RAGE in peripheral nerve regeneration also suggest that care must be taken when blocking RAGE signaling.

## References

[1] Hebert LE, Scherr PA, Bienias JL, Bennett DA, Evans DA (2003). Alzheimer disease in the US population: prevalence estimates using the 2000 census. *Archives of Neurology*.

[2] Muller-Hill B, Beyreuther K (1989). Molecular biology of Alzheimer’s disease. *Annual Review of Biochemistry*.

[3] Glenner GG, Wong CW (1984). Alzheimer’s disease: initial report of the purification and characterization of a novel cerebrovascular amyloid protein. *Biochemical and Biophysical Research Communications*.

[4] Lovell MA, Robertson JD, Teesdale WJ, Campbell JL, Markesbery WR (1998). Copper, iron and zinc in Alzheimer’s disease senile plaques. *Journal of the Neurological Sciences*.

[5] Kosik KS, Joachim CL, Selkoe DJ (1986). Microtubule-associated protein tau (tau) is a major antigenic component of paired helical filaments in Alzheimer disease. *Proceedings of the National Academy of Sciences of the United States of America*.

[6] Thies E, Mandelkow E-M (2007). Missorting of tau in neurons causes degeneration of synapses that can be rescued by the kinase MARK2/Par-1. *Journal of Neuroscience*.

[7] Vega IE, Traverso EE, Ferrer-Acosta Y (2008). A novel calcium-binding protein is associated with tau proteins in tauopathy. *Journal of Neurochemistry*.

[8] Supnet C, Bezprozvanny I (2010). The dysregulation of intracellular calcium in Alzheimer disease. *Cell Calcium*.

[9] Mrak RE, Griffin WST (2001). The role of activated astrocytes and of the neurotrophic cytokine S100B in the pathogenesis of Alzheimer’s disease. *Neurobiology of Aging*.

[10] Boom A, Pochet R, Authelet M (2004). Astrocytic calcium/zinc binding protein S100A6 over expression in Alzheimer’s disease and in PS1/APP transgenic mice models. *Biochimica et Biophysica Acta*.

[11] Akiyama H, Ikeda K, Katoh M, McGeer EG, McGeer PL (1994). Expression of MRP14, 27E10, interferon-*α* and leukocyte common antigen by reactive microglia in postmortem human brain tissue. *Journal of Neuroimmunology*.

[12] Shepherd CE, Goyette J, Utter V (2006). Inflammatory S100A9 and S100A12 proteins in Alzheimer’s disease. *Neurobiology of Aging*.

[13] Yan SD, Chen X, Fu J (1996). RAGE and amyloid-*β* peptide neurotoxicity in Alzheimer’s disease. *Nature*.

[14] Miller MC, Tavares R, Johanson CE (2008). Hippocampal RAGE immunoreactivity in early and advanced Alzheimer’s disease. *Brain Research*.

[15] Schmidt AM, Vianna M, Gerlach M (1992). Isolation and characterization of two binding proteins for advanced glycosylation end products from bovine lung which are present on the endothelial cell surface. *The Journal of Biological Chemistry*.

[16] Neeper M, Schmidt AM, Brett J (1992). Cloning and expression of a cell surface receptor for advanced glycosylation end products of proteins. *The Journal of Biological Chemistry*.

[17] Ahmed N, Ahmed U, Thornalley PJ, Hager K, Fleischer G, Münch G (2005). Protein glycation, oxidation and nitration adduct residues and free adducts of cerebrospinal fluid in Alzheimer’s disease and link to cognitive impairment. *Journal of Neurochemistry*.

[18] Li J, Schmidt AM (1997). Characterization and functional analysis of the promoter of RAGE, the receptor for advanced glycation end products. *The Journal of Biological Chemistry*.

[19] Bierhaus A, Schiekofer S, Schwaninger M (2001). Diabetes-associated sustained activation of the transcription factor nuclear factor-*κ*B. *Diabetes*.

[20] Schmidt A-M, Hofmann M, Taguchi A, Yan SD, Stern DM (2000). RAGE: a multiligand receptor contributing to the cellular response in diabetic vasculopathy and inflammation. *Seminars in Thrombosis and Hemostasis*.

[21] Dickson DW, Sinicropi S, Yen S-H (1996). Glycation and microglial reaction in lesions of Alzheimer’s disease. *Neurobiology of Aging*.

[22] Sasaki N, Fukatsu R, Tsuzuki K (1998). Advanced glycation end products in Alzheimer’s disease and other neurodegenerative diseases. *American Journal of Pathology*.

[23] Smith MA, Taneda S, Richey PL (1994). Advanced Maillard reaction end products are associated with Alzheimer disease pathology. *Proceedings of the National Academy of Sciences of the United States of America*.

[24] Sousa MM, Yan SD, Stern D, Saraiva MJ (2000). Interaction of the receptor for advanced glycation end products (RAGE) with transthyretin triggers nuclear transcription factor kB (NF-kB) activation. *Laboratory Investigation*.

[25] Stürchler E, Galichet A, Weibel M, Leclerc E, Heizmann CW (2008). Site-specific blockade of RAGE-Vd prevents amyloid-*β* oligomer neurotoxicity. *Journal of Neuroscience*.

[26] Takuma K, Fang F, Zhang W (2010). RAGE-mediated signaling contributes to intraneuronal transport of amyloid-*β* and neuronal dysfunction. *Proceedings of the National Academy of Sciences of the United States of America*.

[27] Deane R, Yan SD, Submamaryan RK (2003). RAGE mediates amyloid-*β* peptide transport across the blood-brain barrier and accumulation in brain. *Nature Medicine*.

[28] Buxbaum JN, Ye Z, Reixach N (2008). Transthyretin protects Alzheimer’s mice from the behavioral and biochemical effects of A*β* toxicity. *Proceedings of the National Academy of Sciences of the United States of America*.

[29] Hori O, Brett J, Slattery T (1995). The receptor for advanced glycation end products (RAGE) is a cellular binding site for amphoterin. Mediation of neurite outgrowth and co-expression of RAGE and amphoterin in the developing nervous system. *The Journal of Biological Chemistry*.

[30] Sims GP, Rowe DC, Rietdijk ST, Herbst R, Coyle AJ (2010). HMGB1 and RAGE in inflammation and cancer. *Annual Review of Immunology*.

[31] Heizmann CW, Ackermann GE, Galichet A (2007). Pathologies involving the S100 proteins and RAGE. *Calcium Signalling and Disease*.

[32] Donato R (2003). Intracellular and extracellular roles of S100 proteins. *Microscopy Research and Technique*.

[33] Zimmer DB, Wright Sadosky P, Weber DJ (2003). Molecular mechanisms of S100-target protein interactions. *Microscopy Research and Technique*.

[34] Marenholz I, Lovering RC, Heizmann CW (2006). An update of the S100 nomenclature. *Biochimica et Biophysica Acta*.

[35] Sugaya K, Fukagawa T, Matsumoto K-I (1994). Three genes in the human MHC class III region near the junction with the class II: gene for receptor of advanced glycosylation end products, PBX2 homeobox gene and a notch homolog, human counterpart of mouse mammary tumor gene int-3. *Genomics*.

[36] Hudson BI, Carter AM, Harja E (2008). Identification, classification, and expression of RAGE gene splice variants. *The FASEB Journal*.

[37] Srikrishna G, Huttunen HJ, Johansson L (2002). *N*-glycans on the receptor for advanced glycation end products influence amphoterin binding and neurite outgrowth. *Journal of Neurochemistry*.

[38] Wilton R, Yousef MA, Saxena P, Szpunar M, Stevens FJ (2006). Expression and purification of recombinant human receptor for advanced glycation endproducts in *Escherichia coli*. *Protein Expression and Purification*.

[39] Osawa M, Yamamoto Y, Munesue S (2007). De-*N*-glycosylation or G82S mutation of RAGE sensitizes its interaction with advanced glycation endproducts. *Biochimica et Biophysica Acta*.

[40] Malherbe P, Richards JG, Gaillard H (1999). cDNA cloning of a novel secreted isoform of the human receptor for advanced glycation end products and characterization of cells co-expressing cell-surface scavenger receptors and Swedish mutant amyloid precursor protein. *Molecular Brain Research*.

[41] Yonekura H, Yamamoto Y, Sakurai S (2003). Novel splice variants of the receptor for advanced glycation end-products expressed in human vascular endothelial cells and pericytes, and their putative roles in diabetes-induced vascular injury. *Biochemical Journal*.

[42] Schlueter C, Hauke S, Flohr AM, Rogalla P, Bullerdiek J (2003). Tissue-specific expression patterns of the RAGE receptor and its soluble forms—a result of regulated alternative splicing?. *Biochimica et Biophysica Acta*.

[43] Buckley ST, Ehrhardt C (2010). The receptor for advanced glycation end products (RAGE) and the lung. *Journal of Biomedicine & Biotechnology*.

[44] Ding Q, Keller JN (2005). Splice variants of the receptor for advanced glycosylation end products (RAGE) in human brain. *Neuroscience Letters*.

[45] Galichet A, Weibel M, Heizmann CW (2008). Calcium-regulated intramembrane proteolysis of the RAGE receptor. *Biochemical and Biophysical Research Communications*.

[46] Raucci A, Cugusi S, Antonelli A (2008). A soluble form of the receptor for advanced glycation endproducts (RAGE) is produced by proteolytic cleavage of the membrane-bound form by the sheddase a disintegrin and metalloprotease 10 (ADAM10). *The FASEB Journal*.

[47] Zhang L, Bukulin M, Kojro E (2008). Receptor for advanced glycation end products is subjected to protein ectodomain shedding by metalloproteinases. *The Journal of Biological Chemistry*.

[48] Emanuele E, D’Angelo A, Tomaino C (2005). Circulating levels of soluble receptor for advanced glycation end products in Alzheimer disease and vascular dementia. *Archives of Neurology*.

[49] Nozaki I, Watanabe T, Kawaguchi M (2007). Reduced expression of endogenous secretory receptor for advanced glycation endproducts in hippocampal neurons of Alzheimer’s disease brains. *Archives of Histology and Cytology*.

[50] Kojro E, Postina R (2009). Regulated proteolysis of RAGE and AbetaPP as possible link between type 2 diabetes mellitus and Alzheimer’s disease. *Journal of Alzheimer’s Disease*.

[51] Koyama H, Yamamoto H, Nishizawa Y (2007). RAGE and soluble RAGE: potential therapeutic targets for cardiovascular diseases. *Molecular Medicine*.

[52] Santilli F, Vazzana N, Bucciarelli LG, Davì G (2009). Soluble forms of RAGE in human diseases: clinical and therapeutical implications. *Current Medicinal Chemistry*.

[53] Tesařová P, Kalousová M, Jáchymová M, Mestek O, Petruzelka L, Zima T (2007). Receptor for advanced glycation end products (RAGE)—soluble form (sRAGE) and gene polymorphisms in patients with breast cancer. *Cancer Investigation*.

[54] Nakamura K, Yamagishi S-I, Adachi H (2008). Serum levels of soluble form of receptor for advanced glycation end products (sRAGE) are positively associated with circulating AGEs and soluble form of VCAM-1 in patients with type 2 diabetes. *Microvascular Research*.

[55] Bierhaus A, Nawroth PP (2009). Multiple levels of regulation determine the role of the receptor for AGE (RAGE) as common soil in inflammation, immune responses and diabetes mellitus and its complications. *Diabetologia*.

[56] Ling LR, Gooch C, Szabolcs M (2005). RAGE: a journey from the complications of diabetes to disorders of the nervous system—striking a fine balance between injury and repair. *Restorative Neurology and Neuroscience*.

[57] Ling LR, Trojaborg W, Qu W (2004). Antagonism of RAGE suppresses peripheral nerve regeneration. *The FASEB Journal*.

[58] Rong LL, Yan S-F, Wendt T (2004). RAGE modulates peripheral nerve regeneration via recruitment of both inflammatory and axonal outgrowth pathways. *The FASEB Journal*.

[59] Hofmann MA, Drury S, Fu C (1999). RAGE mediates a novel proinflammatory axis: a central cell surface receptor for S100/calgranulin polypeptides. *Cell*.

[60] Dattilo BM, Fritz G, Leclerc E, Vander Kooi CW, Heizmann CW, Chazin WJ (2007). The extracellular region of the receptor for advanced glycation end products is composed of two independent structural units. *Biochemistry*.

[61] Ostendorp T, Leclerc E, Galichet A (2007). Structural and functional insights into RAGE activation by multimeric S100B. *The EMBO Journal*.

[62] Leclerc E, Fritz G, Weibel M, Heizmann CW, Galichet A (2007). S100B and S100A6 differentially modulate cell survival by interacting with distinct RAGE (receptor for advanced glycation end products) immunoglobulin domains. *The Journal of Biological Chemistry*.

[63] Xie J, Reverdatto S, Frolov A, Hoffmann R, Burz DS, Shekhtman A (2008). Structural basis for pattern recognition by the receptor for advanced glycation end products (RAGE). *The Journal of Biological Chemistry*.

[64] Kislinger T, Fu C, Huber B (1999). *N*
^*ε*^-(carboxymethyl)lysine adducts of proteins are ligands for receptor for advanced glycation end products that activate cell signaling pathways and modulate gene expression. *The Journal of Biological Chemistry*.

[65] Uetz-Von Allmen E, Koch M, Fritz G, Legler DF (2008). V domain of RAGE interacts with AGEs on prostate carcinoma cells. *Prostate*.

[66] Monteiro FA, Cardoso I, Sousa MM, Saraiva MJ (2006). In vitro inhibition of transthyretin aggregate-induced cytotoxicity by full and peptide derived forms of the soluble receptor for advanced glycation end products (RAGE). *FEBS Letters*.

[67] Thinakaran G, Koo EH (2008). Amyloid precursor protein trafficking, processing, and function. *The Journal of Biological Chemistry*.

[68] Klein WL (2002). A*β* toxicity in Alzheimer’s disease: globular oligomers (ADDLs) as new vaccine and drug targets. *Neurochemistry International*.

[69] Dahlgren KN, Manelli AM, Blaine Stine W, Baker LK, Krafft GA, Ladu MJ (2002). Oligomeric and fibrillar species of amyloid-*β* peptides differentially affect neuronal viability. *The Journal of Biological Chemistry*.

[70] McLean CA, Cherny RA, Fraser FW (1999). Soluble pool of A*β* amyloid as a determinant of severity of neurodegeneration in Alzheimer’s disease. *Annals of Neurology*.

[71] Cleary JP, Walsh DM, Hofmeister JJ (2005). Natural oligomers of the amyloid-*β* protein specifically disrupt cognitive function. *Nature Neuroscience*.

[72] Walsh DM, Klyubin I, Fadeeva JV (2002). Naturally secreted oligomers of amyloid *β* protein potently inhibit hippocampal long-term potentiation in vivo. *Nature*.

[73] Lambert MP, Barlow AK, Chromy BA (1998). Diffusible, nonfibrillar ligands derived from A*β*1-42 are potent central nervous system neurotoxins. *Proceedings of the National Academy of Sciences of the United States of America*.

[74] Huang THJ, Yang D-S, Fraser PE, Chakrabartty A (2000). Alternate aggregation pathways of the Alzheimer *β*-amyloid peptide: an *in vitro* model of preamyloid. *The Journal of Biological Chemistry*.

[75] Walsh DM, Klyubin I, Fadeeva JV, Rowan MJ, Selkoe DJ (2002). Amyloid-*β* oligomers: their production, toxicity and therapeutic inhibition. *Biochemical Society Transactions*.

[76] Hartley DM, Walsh DM, Ye CP (1999). Protofibrillar intermediates of amyloid *β*-protein induce acute electrophysiological changes and progressive neurotoxicity in cortical neurons. *Journal of Neuroscience*.

[77] Deshpande A, Mina E, Glabe C, Busciglio J (2006). Different conformations of amyloid *β* induce neurotoxicity by distinct mechanisms in human cortical neurons. *Journal of Neuroscience*.

[78] Wogulis M, Wright S, Cunningham D, Chilcote T, Powell K, Rydel RE (2005). Nucleation-dependent polymerization is an essential component of amyloid-mediated neuronal cell death. *Journal of Neuroscience*.

[79] Moreira PI, Duarte AI, Santos MS, Rego AC, Oliveira CR (2009). An integrative view of the role of oxidative stress, mitochondria and insulin in Alzheimer’s disease. *Journal of Alzheimer’s Disease*.

[80] Reddy VP, Zhu X, Perry G, Smith MA (2009). Oxidative stress in diabetes and Alzheimer’s disease. *Journal of Alzheimer’s Disease*.

[81] Hadding A, Kaltschmidt B, Kaltschmidt C (2004). Overexpression of receptor of advanced glycation end products hypersensitizes cells for amyloid beta peptide-induced cell death. *Biochimica et Biophysica Acta*.

[82] Hsia AY, Masliah E, Mcconlogue L (1999). Plaque-independent disruption of neural circuits in Alzheimer’s disease mouse models. *Proceedings of the National Academy of Sciences of the United States of America*.

[83] Arancio O, Zhang HP, Chen X (2004). RAGE potentiates A*β*-induced perturbation of neuronal function in transgenic mice. *The EMBO Journal*.

[84] Vodopivec I, Galichet A, Knobloch M, Bierhaus A, Heizmann CW, Nitsch RM (2009). RAGE does not affect amyloid pathology in transgenic arcA*β* mice. *Neurodegenerative Diseases*.

[85] Knobloch M, Farinelli M, Konietzko U, Nitsch RM, Mansuy IM (2007). A*β* oligomer-mediated long-term potentiation impairment involves protein phosphatase 1-dependent mechanisms. *Journal of Neuroscience*.

[86] Dearie R, Sagare A, Zlokovic BV (2008). The role of the cell surface LRP and soluble LRP in blood-brain barrier A*β* clearance in Alzheimer’s disease. *Current Pharmaceutical Design*.

[87] Moore BW (1965). A soluble protein characteristic of the nervous system. *Biochemical and Biophysical Research Communications*.

[88] Bell K, Shokrian D, Potenzieri C, Whitaker-Azmitia PM (2003). Harm avoidance, anxiety, and response to novelty in the adolescent S-100*β* transgenic mouse: role of serotonin and relevance to down syndrome. *Neuropsychopharmacology*.

[89] Nishiyama H, Takemura M, Takeda T, Itohara S (2002). Normal development of serotonergic neurons in mice lacking S100B. *Neuroscience Letters*.

[90] Dyck RH, Bogoch II, Marks A, Melvin NR, Teskey GC (2002). Enhanced epileptogenesis in S100B knockout mice. *Molecular Brain Research*.

[91] Schaub MC, Heizmann CW (2008). Calcium, troponin, calmodulin, S100 proteins: from myocardial basics to new therapeutic strategies. *Biochemical and Biophysical Research Communications*.

[92] Marenholz I, Heizmann CW, Fritz G (2004). S100 proteins in mouse and man: from evolution to function and pathology (including an update of the nomenclature). *Biochemical and Biophysical Research Communications*.

[93] Krebs J, Heizmann CW, Krebs J, Michalak M (2007). Calcium-binding proteins and the EF-hand principle. *Calcium: A Matter of Life or Death*.

[94] Gifford JL, Walsh MP, Vogel HJ (2007). Structures and metal-ion-binding properties of the Ca^2+^-binding helix-loop-helix EF-hand motifs. *Biochemical Journal*.

[95] Baudier J, Glasser N, Gerard D (1986). Ions binding to S100 proteins. I. Calcium- and zinc-binding properties of bovine brain S100 alpha alpha, S100a (alpha beta), and S100b (beta beta) protein: Zn^2+^ regulates Ca^2+^ binding on S100b protein. *The Journal of Biological Chemistry*.

[96] Fritz G, Heizmann CW, Messerschmidt A, Bode W, Cygler M (2004). 3D structures of the calcium and zinc binding S100 proteins. *Handbook of Metalloproteins*.

[97] Senior SZ, Mans LL, VanGuilder HD, Kelly KA, Hendrich MP, Elgren TE (2003). Catecholase activity associated with copper-S100B. *Biochemistry*.

[98] Heizmann CW, Fritz G, Schäfer BW (2002). S100 proteins: structure, functions and pathology. *Frontiers in Bioscience*.

[99] Nishikawa T, Lee ISM, Shiraishi N, Ishikawa T, Ohta Y, Nishikimi M (1997). Identification of S100b protein as copper-binding protein and its suppression of copper-induced cell damage. *The Journal of Biological Chemistry*.

[100] Donato R, Sorci G, Riuzzi F (2009). S100B’s double life: intracellular regulator and extracellular signal. *Biochimica et Biophysica Acta*.

[101] Santamaria-Kisiel L, Rintala-Dempsey AC, Shaw GS (2006). Calcium-dependent and -independent interactions of the S100 protein family. *Biochemical Journal*.

[102] Moroz OV, Dodson GG, Wilson KS, Lukanidin E, Bronstein IB (2003). Multiple structural states of S100A12: a key to its functional diversity. *Microscopy Research and Technique*.

[103] Leukert N, Vogl T, Strupat K, Reichelt R, Sorg C, Roth J (2006). Calcium-dependent tetramer formation of S100A8 and S100A9 is essential for biological activity. *Journal of Molecular Biology*.

[104] Kizawa K, Takahara H, Troxler H, Kleinert P, Mochida U, Heizmann CW (2008). Specific citrullination causes assembly of a globular S100A_3_ homotetramer: a putative Ca^2+^ modulator matures human hair cuticle. *The Journal of Biological Chemistry*.

[105] Foell D, Wittkowski H, Vogl T, Roth J (2007). S100 proteins expressed in phagocytes: a novel group of damage-associated molecular pattern molecules. *Journal of Leukocyte Biology*.

[106] Zimmer DB, Van Eldik LJ (1989). Analysis of the calcium-modulated proteins, S100 and calmodulin, and their target proteins during C6 glioma cell differentiation. *Journal of Cell Biology*.

[107] Davey GE, Murmann P, Heizmann CW (2001). Intracellular Ca^2+^ and Zn^2+^ levels regulate the alternative cell density-dependent secretion of S100B in human glioblastoma cells. *The Journal of Biological Chemistry*.

[108] Rothermundt M, Peters M, Prehn JH, Arolt V (2003). S100B in brain damage and neurodegeneration. *Microscopy Research and Technique*.

[109] Rothermundt M, Ponath G, Arolt V (2004). S100B in schizophrenic psychosis. *International Review of Neurobiology*.

[110] Hauschild A, Engel G, Brenner W (1999). S100B protein detection in serum is a significant prognostic factor in metastatic melanoma. *Oncology*.

[111] Torabian S, Kashani-Sabet M (2005). Biomarkers for melanoma. *Current Opinion in Oncology*.

[112] Van Eldik LJ, Zimmer DB (1987). Secretion of S-100 from rat C6 glioma cells. *Brain Research*.

[113] Gerlach R, Demel G, König H-G (2006). Active secretion of S100B from astrocytes during metabolic stress. *Neuroscience*.

[114] Ciccarelli R, Di Iorio P, Bruno V (1999). Activation of A_1_ adenosine or mGlu3 metabotropic glutamate receptors enhances the release of nerve growth factor and S-100beta protein from cultured astrocytes. *Glia*.

[115] Whitaker-Azmitia PM, Murphy R, Azmitia EC (1990). Stimulation of astroglial 5-HT1A receptors releases the serotonergic growth factor, protein S-100, and alters astroglial morphology. *Brain Research*.

[116] Edwards MM, Robinson SR (2006). TNF alpha affects the expression of GFAP and S100B: implications for Alzheimer’s disease. *Journal of Neural Transmission*.

[117] de Souza DF, Leite MC, Quincozes-Santos A (2009). S100B secretion is stimulated by IL-1*β* in glial cultures and hippocampal slices of rats: likely involvement of MAPK pathway. *Journal of Neuroimmunology*.

[118] Peña LA, Brecher CW, Marshak DR (1995). *β*-amyloid regulates gene expression of glial trophic substance S100*β* in C6 glioma and primary astrocyte cultures. *Molecular Brain Research*.

[119] Iuvone T, Esposito G, De Filippis D (2007). Cannabinoid CB_1_ receptor stimulation affords neuroprotection in MPTP-induced neurotoxicity by attenuating S100B up-regulation in vitro. *Journal of Molecular Medicine*.

[120] Pinto SS, Gottfried C, Mendez A (2000). Immunocontent and secretion of S100B in astrocyte cultures from different brain regions in relation to morphology. *FEBS Letters*.

[121] de Almeida LM, Piñeiro CC, Leite MC (2007). Resveratrol increases glutamate uptake, glutathione content, and S100B secretion in cortical astrocyte cultures. *Cellular and Molecular Neurobiology*.

[122] Abib RT, Quincozes-Santos A, Nardin P (2008). Epicatechin gallate increases glutamate uptake and S100B secretion in C6 cell lineage. *Molecular and Cellular Biochemistry*.

[123] Kligman D, Marshak DR (1985). Purification and characterization of a neurite extension factor from bovine brain. *Proceedings of the National Academy of Sciences of the United States of America*.

[124] Selinfreund RH, Barger SW, Pledger WJ, Van Eldik LJ (1991). Neurotrophic protein S100*β* stimulates glial cell proliferation. *Proceedings of the National Academy of Sciences of the United States of America*.

[125] Tramontina F, Conte S, Gonçalves D (2002). Developmental changes in S100B content in brain tissue, cerebrospinal fluid, and astrocyte cultures of rats. *Cellular and Molecular Neurobiology*.

[126] Huttunen HJ, Kuja-Panula J, Sorci G, Agneletti AL, Donato R, Rauvala H (2000). Coregulation of neurite outgrowth and cell survival by amphoterin and S100 proteins through receptor for advanced glycation end products (RAGE) activation. *The Journal of Biological Chemistry*.

[127] Kögel D, Peters M, König H-G (2004). S100B potently activates p65/c-Rel transcriptional complexes in hippocampal neurons: clinical implications for the role of S100B in excitotoxic brain injury. *Neuroscience*.

[128] Reali C, Scintu F, Pillai R, Donato R, Michetti F, Sogos V (2005). S100B counteracts effects of the neurotoxicant trimethyltin on astrocytes and microglia. *Journal of Neuroscience Research*.

[129] Businaro R, Leone S, Fabrizi C (2006). S100B protects LAN-5 neuroblastoma cells against A*β* amyloid-induced neurotoxicity via RAGE engagement at low doses but increases A*β* amyloid neurotoxicity at high doses. *Journal of Neuroscience Research*.

[130] Ponath G, Schettler C, Kaestner F (2007). Autocrine S100B effects on astrocytes are mediated via RAGE. *Journal of Neuroimmunology*.

[131] Feng L, Matsumoto C, Schwartz A, Schmidt AM, Stern DM, Pile-Spellman J (2005). Chronic vascular inflammation in patients with type 2 diabetes: endothelial biopsy and RT-PCR analysis. *Diabetes Care*.

[132] Man S, Ubogu EE, Ransohoff RM (2007). Inflammatory cell migration into the central nervous system: a few new twists on an old tale. *Brain Pathology*.

[133] Marshak DR, Pesce SA, Stanley LC, Griffin WST (1992). Increased S100*β* neurotrophic activity in Alzheimer’s disease temporal lobe. *Neurobiology of Aging*.

[134] Peskind ER, Griffin WST, Akama KT, Raskind MA, Van Eldik LJ (2001). Cerebrospinal fluid S100B is elevated in the earlier stages of Alzheimer’s disease. *Neurochemistry International*.

[135] Chaves ML, Camozzato AL, Ferreira ED (2010). Serum levels of S100B and NSE proteins in Alzheimer’s disease patients. *Journal of Neuroinflammation*.

[136] Griffin WST, Sheng JG, Mckenzie JE (1998). Life-long overexpression of S100*β* in Down’s syndrome: implications for Alzheimer pathogenesis. *Neurobiology of Aging*.

[137] Petzold A, Jenkins R, Watt HC (2003). Cerebrospinal fluid S100B correlates with brain atrophy in Alzheimer’s disease. *Neuroscience Letters*.

[138] Craft JM, Watterson DM, Marks A, Van Eldik LJ (2005). Enhanced susceptibility of S-100B transgenic mice to neuroinflammation and neuronal dysfunction induced by intracerebroventricular infusion of human *β*-amyloid. *Glia*.

[139] Mori T, Koyama N, Arendash GW, Horikoshi-Sakuraba Y, Tan J, Town T (2010). Overexpression of human S100B exacerbates cerebral amyloidosis and gliosis in the Tg2576 mouse model of Alzheimer’s disease. *Glia*.

[140] Baudier J, Cole RD (1988). Interactions between the microtubule-associated tau proteins and S100B regulate tau phosphorylation by the Ca^2+^/calmodulin-dependent protein kinase II. *The Journal of Biological Chemistry*.

[141] Esposito G, Scuderi C, Lu J (2008). S100B induces tau protein hyperphosphorylation via Dickopff-1 up-regulation and disrupts the Wnt pathway in human neural stem cells. *Journal of Cellular and Molecular Medicine*.

[142] Mruthinti S, Sood A, Humphrey CL, Swamy-Mruthinti S, Buccafusco JJ (2006). The induction of surface *β*-amyloid binding proteins and enhanced cytotoxicity in cultured PC-12 and IMR-32 cells by advanced glycation end products. *Neuroscience*.

[143] Gasic-Milenkovic J, Dukic-Stefanovic S, Deuther-Conrad W, Gärtner U, Münch G (2003). *β*-amyloid peptide potentiates inflammatory responses induced by lipopolysaccharide, interferon -gamma and ’advanced glycation endproducts’ in a murine microglia cell line. *European Journal of Neuroscience*.

[144] Perrone L, Peluso G, Melone MAB (2008). RAGE recycles at the plasma membrane in S100B secretory vesicles and promotes Schwann cells morphological changes. *Journal of Cellular Physiology*.

[145] Hsieh H-L, Schäfer BW, Weigle B, Heizmann CW (2004). S100 protein translocation in response to extracellular S100 is mediated by receptor for advanced glycation endproducts in human endothelial cells. *Biochemical and Biophysical Research Communications*.

[146] Boyd JH, Kan B, Roberts H, Wang Y, Walley KR (2008). S100A8 and S100A9 mediate endotoxin-induced cardiomyocyte dysfunction via the receptor for advanced glycation end products. *Circulation Research*.

[147] Ghavami S, Rashedi I, Dattilo BM (2008). S100A8/A9 at low concentration promotes tumor cell growth via RAGE ligation and MAP kinase-dependent pathway. *Journal of Leukocyte Biology*.

[148] Schmidt AM, Yan SD, Yan SF, Stern DM (2001). The multiligand receptor RAGE as a progression factor amplifying immune and inflammatory responses. *Journal of Clinical Investigation*.

[149] Westermark P, Benson MD, Buxbaum JN (2007). A primer of amyloid nomenclature. *Amyloid*.

[150] Sousa MM, Yan SD, Fernandas R, Guimarães A, Stern D, Saraiva MJ (2001). Familial amyloid polyneuropathy: receptor for advanced glycation end products-dependent triggering of neuronal inflammatory and apoptotic pathways. *Journal of Neuroscience*.

[151] Schwarzman AL, Tsiper M, Wente H (2004). Amyloidogenic and anti-amyloidogenic properties of recombinant transthyretin variants. *Amyloid*.

[152] Link CD (1995). Expression of human *β*-amyloid peptide in transgenic *Caenorhabditis elegans*. *Proceedings of the National Academy of Sciences of the United States of America*.

[153] Stein TD, Anders NJ, DeCarli C, Chan SL, Mattson MP, Johnson JA (2004). Neutralization of transthyretin reverses the neuroprotective effects of secreted amyloid precursor protein (APP) inAPP_Sw_ mice resulting in tau phosphorylation and loss of hippocampal neurons: support for the amyloid hypothesis. *Journal of Neuroscience*.

[154] Ehlermann P, Eggers K, Bierhaus A (2006). Increased proinflammatory endothelial response to S100A8/A9 after preactivation through advanced glycation end products. *Cardiovascular Diabetology*.

[155] Calcutt NA, Cooper ME, Kern TS, Schmidt AM (2009). Therapies for hyperglycaemia-induced diabetic complications: from animal models to clinical trials. *Nature Reviews Drug Discovery*.

[156] Zlokovic BV (2008). New therapeutic targets in the neurovascular pathway in Alzheimer’s disease. *Neurotherapeutics*.

